# Electrical and Thermal Characteristics of AlGaN/GaN HEMT Devices with Dual Metal Gate Structure: A Theoretical Investigation

**DOI:** 10.3390/ma15113818

**Published:** 2022-05-27

**Authors:** Yongfeng Qu, Ningkang Deng, Yuan Yuan, Wenbo Hu, Hongxia Liu, Shengli Wu, Hongxing Wang

**Affiliations:** 1 Key Laboratory for Physical Electronics and Devices of the Ministry of Education, School of Electronic Science and Engineering, Xi’an Jiaotong University, Xi’an 710049, China; yfq0924@163.com (Y.Q.); dnk0309@163.com (N.D.); slwu@mail.xjtu.edu.cn (S.W.); hxwangcn@mail.xjtu.edu.cn (H.W.); 2 Science and Technology on Low-Light-Level Night Vision Laboratory, Xi’an 710065, China; cater2046@163.com; 3 Key Laboratory of Wide Band-Gap Semiconductor Materials and Devices, School of Microelectronics, Xidian University, Xi’an 710071, China; hxliu@mail.xidian.edu.cn

**Keywords:** electrothermal simulation, AlGaN/GaN HEMT, dual-metal gate, self-heating effect

## Abstract

The electrical and thermal characteristics of AlGaN/GaN high-electron mobility transistor (HEMT) devices with a dual-metal gate (DMG) structure are investigated by electrothermal simulation and compared with those of conventional single-metal gate (SMG) structure devices. The simulations reveal that the DMG structure devices have a 10-percent higher transconductance than the SMG structure devices when the self-heating effect is considered. In the meantime, employing the DMG structure, a decrease of more than 11% in the maximum temperature rise of the devices can be achieved at the power density of 6 W/mm. Furthermore, the peak in heat generation distribution at the gate edge of the devices is reduced using this structure. These results could be attributed to the change in the electric field distribution at the gate region and the suppression of the self-heating effect. Therefore, the electrical and thermal performances of AlGaN/GaN HEMT devices are improved by adopting the DMG structure.

## 1. Introduction

Group III-nitride semiconductors represented by gallium nitride (GaN), which have the advantages of a wide bandgap, high-breakdown electric field and high electron mobility, are ideal materials for realizing high-frequency, high-power, and high-voltage electronic devices [1,2,3]. With the rapid development of epitaxial technology, AlGaN/GaN heterostructures are capable of forming two-dimensional electron gas (2DEG) with high concentration and high mobility at the interface due to the polarization effect. Therefore, high-electron mobility transistors (HEMT) based on AlGaN/GaN heterojunctions are widely used in high-frequency and high-power devices [4,5,6].

However, the non-uniform electric field distribution caused by the combined effect of gate and drain voltages has a significant impact on electron motion at the AlGaN/GaN heterojunction interface, resulting in a lower electron velocity on the source side than on the drain side, which lowers the carrier transport efficiency [7]. Related strategies for controlling the electric field distribution, such as field plate technology [8] and a dual-metal gate (DMG) structure [9], have recently been investigated. DMG structures, in particular, can significantly regulate the electric field distribution in the channel, leading to a higher output current in the devices without considering the self-heating effect [10], as well as suppressing short-channel effects such as drain-induced barrier lowering (DIBL) [11]. This gate structure, which is made up of two materials with different work functions, is responsible for improving the electrical performance of the devices. The gate material close to the source has a higher work function than the gate material near the drain. Furthermore, the non-uniform electric field distribution causes a strong localization of Joule heating in AlGaN/GaN HEMT devices, which causes a high-temperature region under the gate edge of the devices (hotspot) [12]. A high hotspot temperature decreases electron mobility and other fundamental properties, reducing the output power, transconductance (*g*_m_), cut-off frequency, maximum oscillation frequency, and reliability of the devices [13]. Therefore, for the development and design of AlGaN/GaN HEMT devices, a comprehensive understanding of the impact of heat generation and temperature rises on the performance of the devices, as well as the relationship with the internal electric field of the devices, is critical. The Joule heating effect of AlGaN/GaN HEMT devices with the single-metal gate (SMG) structure has been widely studied, with experiments [14,15] and simulations [16,17]. In addition, Pinchbeck et al. performed theoretical simulations of the electrical performance of AlGaN/GaN HEMT devices with the DMG structure without considering the self-heating effect. It is suggested that devices with the DMG structure have the advantages of suppressing the DIBL effect and improving electrical performance compared to devices with the SMG structure [18]. Recently, experiments have demonstrated that using the DMG structure improves the electrical performance of AlGaN/GaN HEMT devices [17,19]. However, the self-heating effect of AlGaN/GaN HEMT devices with the DMG structure, which is the main target of this study, has not yet been investigated. Therefore, exploring the physical origin of the self-heating and the internal heat generation mechanism of the DMG structure AlGaN/GaN HEMT devices is essential for the design and development of high-frequency, high-power GaN devices, which will also provide some theoretical guidance for the experimental investigation into the DMG structure of AlGaN/GaN HEMT devices.

In this work, we theoretically investigate the electrical and thermal characteristics of AlGaN/GaN HEMT devices with a DMG structure, and the heat generation mechanism of the devices is explored. The AlGaN/GaN HEMT devices with the DMG structure are analyzed and compared to conventional devices with the SMG structure using electrothermal simulation. The transfer and output characteristics of the devices, as well as the heat generation distribution, channel temperature, and electric field distribution inside the devices, are studied to evaluate the overall performance of the DMG structure GaN/AlGaN HEMT devices.

## 2. Models and Methods 

**Figure 1:**
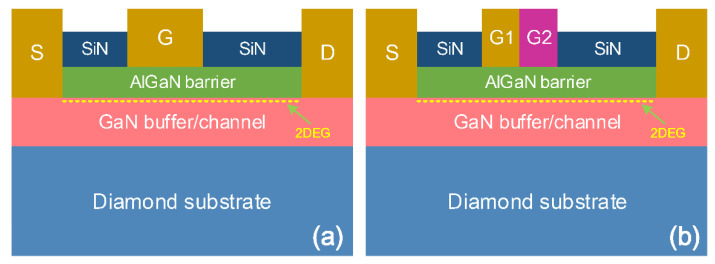
Schematics of AlGaN/GaN HEMT devices with (**a**) the SMG structure, and (**b**) the DMG structure, respectively.

To investigate the electrical and thermal characteristics of AlGaN/GaN HEMT devices with DMG structure, an electro-thermal model is established, and a systematic comparison with AlGaN/GaN HEMT devices with an SMG structure is performed. Figure 1 illustrates the cross-sectional schematics of AlGaN/GaN HEMT device layouts with DMG and SMG structures, respectively. The AlGaN and GaN layers of the devices investigated in this study have thicknesses of *t*_AlGaN_ = 25 nm and *t*_GaN_ = 1.475 μm, respectively. Both AlGaN and GaN layers have an unintentional background doping level of 1 × 10^15^ cm^−3^. The top of the AlGaN layer is passivated with 100 nm of SiN to avoid surface state issues [20]. The total gate length for both devices is *L*_G_ = 1 μm, with *L*_SG_ = 2 μm and *L*_GD_ = 4 μm for gate-source and gate-drain spacing, respectively. The gate metal of the SMG structure devices has a work function of 5.2 eV, whereas the gate metal of the DMG structure devices is comprised of two metals with different work functions of 5.2 and 4.4 eV, respectively. For instance, palladium (Pd) and titanium (Ti) with work function of 5.2 and 4.4 eV can be used as a gate metal for the DMG structure devices. The gate metal with a higher work function near the source is referred to as Gate 1 and the gate metal with a lower work function near the drain is referred to as Gate 2. The gate lengths of both Gate 1 and Gate 2 are 0.5 μm. The detailed fabrication steps for the proposed AlGaN/GaN HEMT devices with DMG structure can be found in the previous literature [18]. Moreover the most common method of realizing the DMG structure AlGaN/GaN HEMT on diamond substrate is the diamond substrate transfer technique [21].

A commercial ATLAS TCAD device simulator was used to perform electrothermal simulations of AlGaN/GaN HEMT devices with the SMG and DMG configurations [22]. The material parameters used in the simulation are given in Table 1. The electron and heat transport equations were solved simultaneously in the simulation using the drift-diffusion model. The simulations consider spontaneous polarization and strain-induced piezoelectric polarization to induce the formation of 2DEG at the AlGaN/GaN interface. The Shockley–Read–Hall model, the high-field-dependent mobility model, and the Farahmand Modified Caughey Thomas (FMCT) mobility model for the low field were also employed in the simulation studies [23].

The boundary conditions for the heat flow calculation assume that the bottom of the substrate is a constant-temperature surface with an ambient temperature of 300 K, while the other exterior surfaces are adiabatic. The thermal boundary resistance (TBR) at the interface between diamond and GaN materials, as well as the temperature-dependent thermal conductivity (*κ*(*T*)), were considered in the model for realistic modeling. The diamond–GaN interface had a TBR of 2.06 × 10^−8^ m^2^K/W [24]. Kirchhoff transform was used to describe the nonlinear thermal conductivity of AlGaN, GaN, and diamond materials [25]:

(1) $$k\left(\mathrm{AlGaN}\right)=25\cdot {\left(\frac{T}{300}\right)}^{-1.44}$$

(2) $$k\left(\mathrm{GaN}\right)=160\cdot {\left(\frac{T}{300}\right)}^{-1.42}$$

(3) $$k\left(\mathrm{Diamond}\right)=1480\cdot {\left(\frac{T}{300}\right)}^{-0.55}$$

## 3. Results and Discussion

**Figure 2:**
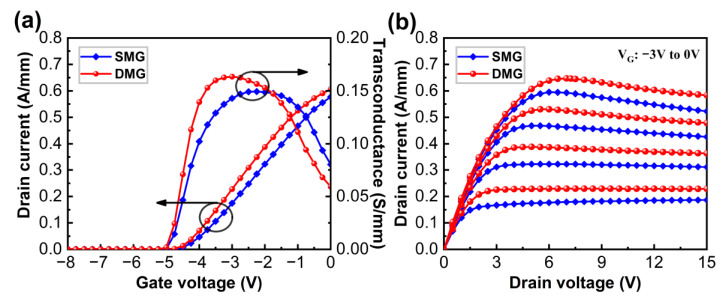
(**a**) *I*_DS_-*V*_GS_ and *g*_m_-*V*_GS_ transfer curves of AlGaN/GaN HEMT devices with SMG and DMG structures at *V*_DS_ = 5 V. (**b**) *I*_DS_-*V*_DS_ characteristics of AlGaN/GaN HEMT devices with SMG and DMG structures at various *V*_GS_ ranging from −3–0 V with a step of 1 V.

The transfer and output characteristics of AlGaN/GaN HEMT devices with the DMG and SMG structures were calculated to better understand the electrothermal behavior of devices with the DMG structure. Figure 2a illustrates the drain current (*I*_DS_) versus gate voltage (*V*_GS_) transfer characteristic and *g*_m_ of AlGaN/GaN HEMT devices for both the DMG and SMG configurations when the drain voltage (*V*_DS_) is 5 V. The threshold voltage (*V*_th_) for both devices with the DMG and SMG structures was about −4.2 V, as can be observed. Moreover, the maximum *g*_m_ value of the DMG structure devices (0.164 S/mm) is 10.0% higher than that of the SMG structure devices (0.149 S/mm), which is consistent with the experimental results reported by Pinchbeck et al. [18] and Visvkarma et al. [19]. Figure 2b shows the *I*_D_-*V*_DS_ output characteristics of the DMG structure and SMG structure devices at various *V*_GS_ ranging from −3 V to 0 V with a step of 1 V. The devices with a DMG structure have a larger saturation output current than the SMG structure devices, which is consistent with the obtained results without accounting for the self-heating effect [11]. When the self-heating effect is taken into account, the saturation output currents of the DMG and SMG structure devices exhibit a drain current degradation as the drain bias voltage increases, especially at a high *V*_GS_. The drain current in the saturation region of both devices is hardly decreased at a low *V*_GS_ due to the higher thermal conductivity of the diamond substrate, which rapidly exports heat from device hotspots. The analysis of the characteristics demonstrates that the DMG structure devices still have a higher *g*_m_ and higher output current than the SMG structure devices when considering the self-heating effect. 

**Figure 3:**
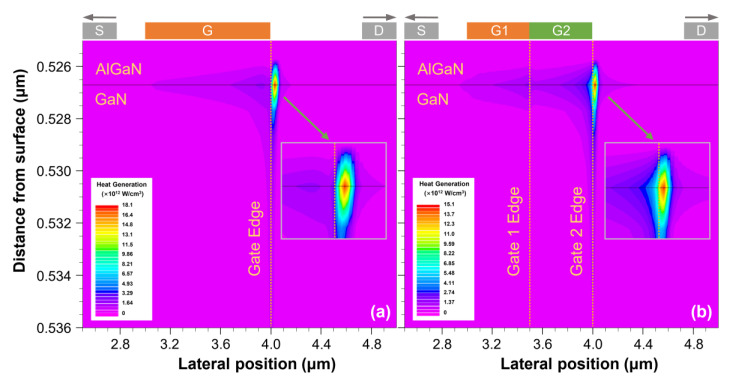
Heat generation profiles of (**a**) the SMG structure and (**b**) the DMG structure AlGaN/GaN HEMT devices at the power density *P* = 6 W/mm.

To further understand the thermal characteristics of AlGaN/GaN HEMT devices, we examined and analyzed the heat generation distribution under the gate of the SMG structure and DMG structure devices at the same power density *P* = 6 W/mm, as shown in Figure 3. To reach *P* = 6 W/mm, the drain voltages of the SMG structure and DMG structure devices are *V*_DS_ = 10.8 V and *V*_DS_ = 9.6 V, respectively, while the gate voltage is set to *V*_GS_ = 0 V. The heat generation distribution of the SMG structure devices illustrated in Figure 3a is highly localized at the gate edge on the drain side of the devices, with a little heat generation distribution in other regions of the 2DEG channel. Figure 3b shows that the heat generation distribution peak of the DMG structure devices is also present at the edge of the gate on the drain side; meanwhile, in comparison to the SMG structure devices, the presence of the DMG causes the hotspot to slightly move toward the source side and a moderate amount of heat generation to spread along the 2DEG channel, resulting in a reduction in heat generation in the hotspot region. The peak heat generations of the SMG and DMG structure devices are 1.80 × 10^13^ and 1.50 × 10^13^ W/cm^3^, respectively. These results indicate that the DMG structure can reduce the peak in heat generation distribution of the devices and enables a greater extension of the heat generation along the 2DEG channel direction. 

**Figure 4:**
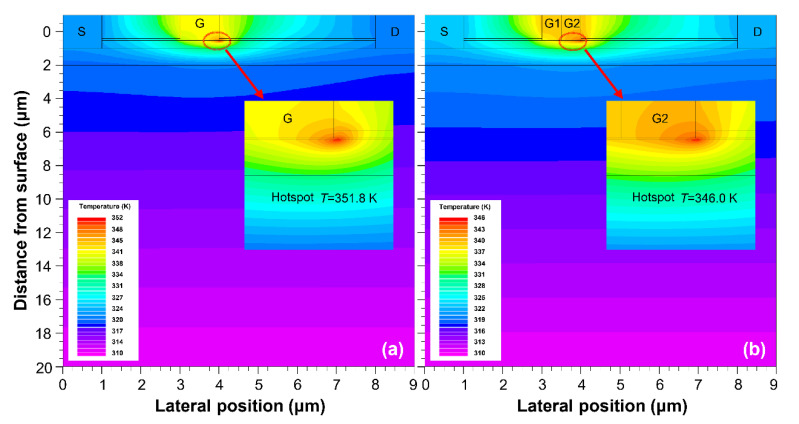
Temperature distribution profiles of AlGaN/GaN HEMT devices with the power density of *P* = 6 W/mm. (**a**) SMG structure. (**b**) DMG structure.

**Figure 5:**
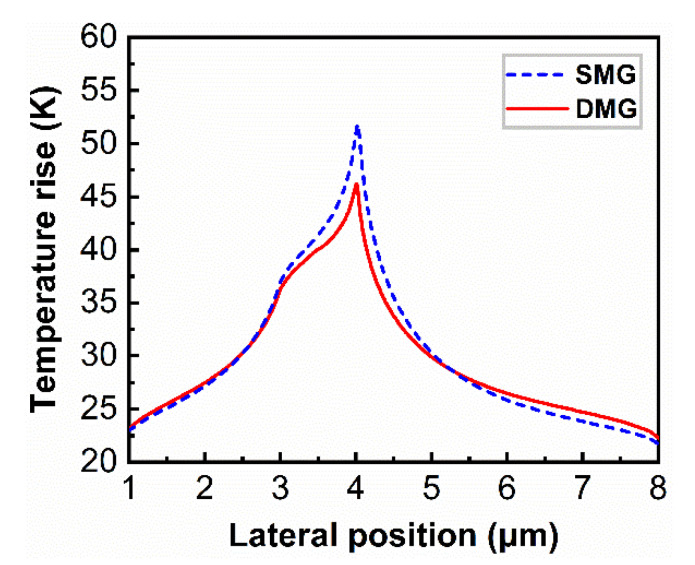
Temperature rise distributions of AlGaN/GaN HEMT devices along the 2DEG channel with the power density of *P* = 6 W/mm.

Figure 4 depicts the temperature distribution profiles of AlGaN/GaN HEMT devices with the SMG and DMG structures at the power dissipation of 6 W/mm. Temperature peaks are located at the gate edges in both devices, and heat propagates along the 2DEG channel and substrate directions. Figure 5 illustrates the temperature rise distribution along the 2DEG channel, which is extracted from the temperature distribution of the SMG and DMG structural devices. The highest temperature rise for the SMG structure devices is 51.8 K, while the temperature rise for the DMG structure is 46.0 K. The peak temperature rise in the DMG structure devices is around 11.2% lower than that of the SMG structure devices. In addition, we estimated the thermal resistance of the SMG and DMG structure devices, which was calculated as (*R*_th_ = (*T*_j_ − *T*_ref_)/(*P*)), where *T*_j_ and *T*_ref_ are the hotspot temperature and substrate bottom surface temperature, respectively. At the same power density (*P* = 6 W/mm), the DMG structure device has lower thermal resistance (*R*_th_ = 7.67 K mm/W) than the SMG structure device (*R*_th_ = 8.63 K mm/W). The lower-temperature peaks contribute to the reliability and electrical characteristics of the devices, and these findings show that the DMG structure devices have better thermal management than the SMG structure devices.

**Figure 6:**
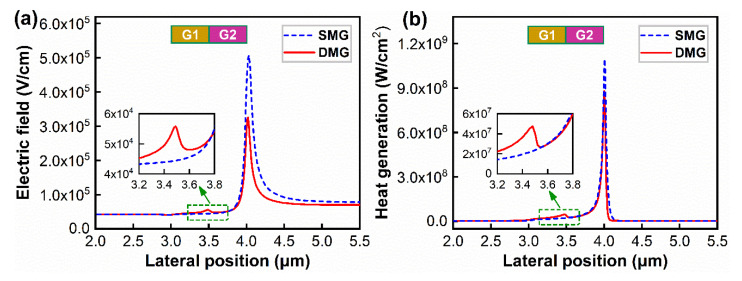
The distribution profiles of (**a**) electric field and (**b**) heat generation of AlGaN/GaN HEMT devices along the 2DEG channel with the power density of *P* = 6 W/mm.

Figure 6 presents the distributions of electric field and heat generation along the 2DEG channel of the SMG and DMG structures devices, which is used to further understand the mechanism of heat generation in the channel of the devices. Figure 6a shows that the SMG structure devices have a strong electric field peak in the channel, but the DMG structure devices have two electric field peaks in the channel. Furthermore, compared to the SMG structure devices, the DMG structure devices have a lower-intensity electric field peak at Gate 1 edge as well as a significant reduction in the intensity of the electric field peak at Gate 2 edge on the drain side. This acts in a similar manner as field plates, spreading out the peak field seen along the device channel [26]. The peak electric field accelerates electrons at the gate edge, leading to very high phonon scattering, which generates a large amount of heat at the gate edge on the drain side, greatly reducing the electron mobility and seriously affecting the electrical performance of the devices. Therefore, the electric field distribution of the DMG structure device helps to reduce the peak temperature at the gate edge and improve the electrical performance of the devices. Moreover, according to the heat generation distribution in the 2DEG channel in Figure 6b, the heat generation peaks of both devices are highly localized at the gate edge on the drain side, while the DMG structure devices have a smaller heat generation peak at Gate 1 edge in addition to a higher heat generation peak at Gate 2 edge, which corresponds to the electric field distribution. Therefore, these results prove that the DMG structure can be used to regulate the electric field and heat generation distribution, reducing the hotspot temperature and suppressing the self-heating effect of AlGaN/GaN HEMT devices.

**Figure 7:**
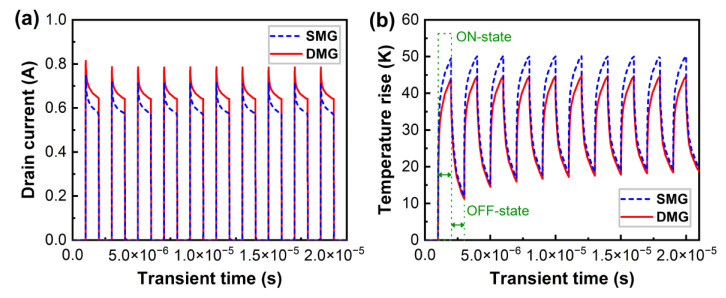
(**a**) Transient drain current of AlGaN/GaN HEMT devices when power density switched from 0–6 W/mm at constant gate voltage (*V*_GS_ = 0 V). (**b**) The transient temperature rise distributions of AlGaN/GaN HEMT devices under pulse simulation.

Device self-heating in switching applications is the duty cycle, pulse period, and other parameters. In addition the reliability of the devices is affected by self-heating and the operating time. As a result, as shown in Figure 7, transient simulations of AlGaN/GaN HEMT devices with the SMG and DMG structures are performed using a power density of 6 W/mm over ten pulse repetition periods. The pulse period is 2 μs and the pulse width is 1 μs. Figure 7a shows that the drain current for all devices decreases with time during one period, and the maximum drain current of the DMG structure devices is greater than that of the SMG structure devices. Transient simulations were also employed to evaluate the channel temperature rise in the SMG and DMG structure devices, as illustrated in Figure 7b. The thermal response is demonstrated to vary rapidly in response to a sudden increase in power owing to Joule heating, with the trend being that the channel temperature first rises instantly and then continues to grow approximately linearly with increasing load power time throughout the ON-state. The channel temperature decreases immediately as the power returns to 0 W at the OFF-state. These results are consistent with the reported results in [27]. At the same time, the thermal response is consistent over pulse repetition cycles, while the channel temperature rise is nearly constant, due to the excellent thermal conductivity of the diamond substrate, which allows for heat to be exported quickly. Furthermore, at the same operating power, the channel temperature rise of the DMG structure devices is lower than that of the SMG structure devices.

## 4. Conclusions

In summary, the electrical and thermal characteristics of AlGaN/GaN HEMT devices on diamond substrates with the DMG structure are investigated by electrothermal simulations and compared with those of the conventional SMG structure devices. The DMG structure can effectively improve the transconductance and output current of the devices. The maximum *g*_m_ value of the DMG devices (0.164 S/mm) was found to be 10.0% higher than that of the SMG devices (0.149 S/mm). In addition, the DMG structure changes the electric field distribution in the channel of the devices, decreasing the peak electric field at Gate 2 edge on the drain side and thus reducing the scattering between phonons and between phonons and electrons in the channel. This result leads to a reduction in the heat generation peak of the devices with the DMG structure. Moreover, the electric field peak at the Gate 1 edge of the devices with the DMG structure contributes to some heat generation extending along the channel and into the GaN buffer layer. AlGaN/GaN HEMT devices using the DMG structure achieve a reduction in maximum temperature rise of over 11% compared to the devices with an SMG structure. These results suggest that the DMG structure effectively improves the electrical characteristics and reduces the hotspot temperature of AlGaN/GaN HEMT devices; thereby, AlGaN/GaN HEMT devices with the DMG structure are a potential candidate for high-power electronics applications.

## Figures and Tables

**Table 1:** Material parameters used in the simulation.

Material Property	AlGaN	GaN	Diamond
Permittivity, ε	8.8	8.9	5.5
Energy band gap, E_g_ (eV)	3.87	3.43	5.47
Electron affinity, χ (eV)	4.01	4.31	1.30
Electron mobility, *μ* (cm^2^/Vs)	300	1200	2000
Saturation velocity, vs. (10^7^ cm/s)	1.1	2.5	1.0
Conduction band state density, N_c_ (10^18^/cm^3^)	2.74	2.24	5.0
Valance band state density, N_v_ (10^19^/cm^3^)	1.98	2.51	1.80

## Data Availability

Not applicable.
